# Supplementary material for: FastqPuri: high-performance preprocessing of RNA-seq data
Source: BMC Bioinformatics. 2019 May 3;20:226. doi: 10.1186/s12859-019-2799-0 (PMC6500068; doi:10.1186/s12859-019-2799-0)
Supplement: Supplementary file 2 — Archive of FastqPuri. Archive containing all files needed to install and run FastqPuri v1.0.6. Date stamp March 22, 2019. (GZ 47,819 kb) [file 12859_2019_2799_MOESM2_ESM.gz › FastqPuri-1.0.6/html/struct__trimFilter_8h_source.html]

FastqPuri: include/struct\_trimFilter.h Source File


FastqPuri


- include

struct\_trimFilter.h

Go to the documentation of this file.

1 /\*\*\*\*\*\*\*\*\*\*\*\*\*\*\*\*\*\*\*\*\*\*\*\*\*\*\*\*\*\*\*\*\*\*\*\*\*\*\*\*\*\*\*\*\*\*\*\*\*\*\*\*\*\*\*\*\*\*\*\*\*\*\*\*\*\*\*\*\*\*\*\*\*\*\*\*

2  \* Copyright (C) 2017 by Paula Perez Rubio \*

3  \* \*

4  \* This file is part of FastqPuri. \*

5  \* \*

6  \* FastqPuri is free software: you can redistribute it and/or modify \*

7  \* it under the terms of the GNU General Public License as \*

8  \* published by the Free Software Foundation, either version 3 of the \*

9  \* License, or (at your option) any later version. \*

10  \* \*

11  \* FastqPuri is distributed in the hope that it will be useful, \*

12  \* but WITHOUT ANY WARRANTY; without even the implied warranty of \*

13  \* MERCHANTABILITY or FITNESS FOR A PARTICULAR PURPOSE. See the \*

14  \* GNU General Public License for more details. \*

15  \* \*

16  \* You should have received a copy of the GNU General Public License \*

17  \* along with FastqPuri. \*

18  \* If not, see <http://www.gnu.org/licenses/>. \*

19  \*\*\*\*\*\*\*\*\*\*\*\*\*\*\*\*\*\*\*\*\*\*\*\*\*\*\*\*\*\*\*\*\*\*\*\*\*\*\*\*\*\*\*\*\*\*\*\*\*\*\*\*\*\*\*\*\*\*\*\*\*\*\*\*\*\*\*\*\*\*\*\*\*\*\*\*/

20

30 #ifndef STRUCT\_TRIMFILTER\_H\_

31 #define STRUCT\_TRIMFILTER\_H\_

32

33 #include "defines.h"

34 #include "bloom.h"

35

39 typedef struct \_adapter {

40  char \*ad\_fa;

41  char \*ad2\_fa;

42  int mismatches;

43  double threshold;

44  int Nad;

45 } Adapter;

46

51 typedef struct \_iparam\_trimFilter {

52  char \*Ifq;

53  char \*Ifq2;

54  char \*Ifa;

55  char \*Iidx;

56  char \*Iinfo;

57  char \*Oprefix;

58  bool uncompress;

59  Adapter ad;

60  Bfkmer \*ptr\_bfkmer;

61  int trimQ;

62  int trimN;

63  int method;

64  bool is\_fa;

65  bool is\_idx;

66  bool is\_adapter;

67  double score;

68  int minQ;

69  int L;

70  int minL;

71  int nlowQ;

72  int kmersize;

73  int globleft;

74  int globright;

75  int percent;

76 } Iparam\_trimFilter;

77

78 void free\_parTF(Iparam\_trimFilter \*ptr\_parTF);

79

80 #endif // endif \_STRUCT\_TRIMFILTER\_H\_

\_iparam\_trimFilter::globright

int globright

**Definition:** struct\_trimFilter.h:74

\_iparam\_trimFilter::ad

Adapter ad

**Definition:** struct\_trimFilter.h:59

free\_parTF

void free\_parTF(Iparam\_trimFilter \*ptr\_parTF)

frees the allocated memory in Iparam\_trimFilter

**Definition:** struct\_trimFilter.c:36

\_iparam\_trimFilter

trimFilter input parameters

**Definition:** struct\_trimFilter.h:51

\_iparam\_trimFilter::Oprefix

char \* Oprefix

**Definition:** struct\_trimFilter.h:57

\_iparam\_trimFilter::minQ

int minQ

**Definition:** struct\_trimFilter.h:68

\_iparam\_trimFilter::method

int method

**Definition:** struct\_trimFilter.h:63

Adapter

struct \_adapter Adapter

bloom.h

functions that implement the bloom filter

\_adapter::mismatches

int mismatches

**Definition:** struct\_trimFilter.h:42

\_iparam\_trimFilter::Iidx

char \* Iidx

**Definition:** struct\_trimFilter.h:55

\_iparam\_trimFilter::Ifa

char \* Ifa

**Definition:** struct\_trimFilter.h:54

\_adapter::ad2\_fa

char \* ad2\_fa

**Definition:** struct\_trimFilter.h:41

\_adapter::threshold

double threshold

**Definition:** struct\_trimFilter.h:43

\_iparam\_trimFilter::Ifq2

char \* Ifq2

**Definition:** struct\_trimFilter.h:53

\_iparam\_trimFilter::score

double score

**Definition:** struct\_trimFilter.h:67

\_iparam\_trimFilter::trimQ

int trimQ

**Definition:** struct\_trimFilter.h:61

\_iparam\_trimFilter::is\_adapter

bool is\_adapter

**Definition:** struct\_trimFilter.h:66

defines.h

Macro definitions.

\_iparam\_trimFilter::globleft

int globleft

**Definition:** struct\_trimFilter.h:73

Iparam\_trimFilter

struct \_iparam\_trimFilter Iparam\_trimFilter

trimFilter input parameters

\_iparam\_trimFilter::trimN

int trimN

**Definition:** struct\_trimFilter.h:62

\_adapter

**Definition:** struct\_trimFilter.h:39

\_iparam\_trimFilter::nlowQ

int nlowQ

**Definition:** struct\_trimFilter.h:71

\_iparam\_trimFilter::kmersize

int kmersize

**Definition:** struct\_trimFilter.h:72

\_iparam\_trimFilter::Ifq

char \* Ifq

**Definition:** struct\_trimFilter.h:52

\_iparam\_trimFilter::L

int L

**Definition:** struct\_trimFilter.h:69

\_bfkmer

stores a processed kmer (2 bits pro nucleotide)

**Definition:** bloom.h:57

\_iparam\_trimFilter::is\_idx

bool is\_idx

**Definition:** struct\_trimFilter.h:65

\_iparam\_trimFilter::minL

int minL

**Definition:** struct\_trimFilter.h:70

\_iparam\_trimFilter::Iinfo

char \* Iinfo

**Definition:** struct\_trimFilter.h:56

\_iparam\_trimFilter::is\_fa

bool is\_fa

**Definition:** struct\_trimFilter.h:64

\_iparam\_trimFilter::ptr\_bfkmer

Bfkmer \* ptr\_bfkmer

**Definition:** struct\_trimFilter.h:60

\_iparam\_trimFilter::uncompress

bool uncompress

**Definition:** struct\_trimFilter.h:58

\_adapter::Nad

int Nad

**Definition:** struct\_trimFilter.h:44

\_adapter::ad\_fa

char \* ad\_fa

**Definition:** struct\_trimFilter.h:40

\_iparam\_trimFilter::percent

int percent

**Definition:** struct\_trimFilter.h:75


---

Generated on Mon Mar 19 2018 23:42:01 for FastqPuri by  

 1.8.14
